# Assessing the burden of *Taenia solium* cysticercosis in Burundi, 2020

**DOI:** 10.1186/s12879-022-07849-7

**Published:** 2022-11-14

**Authors:** Salvator Minani, Brecht Devleesschauwer, Anastasie Gasogo, Jean-Bosco Ntirandekura, Sarah Gabriël, Pierre Dorny, Chiara Trevisan

**Affiliations:** 1 Department of Biology, Faculty of Sciences, University of Burundi, Bujumbura, Burundi; 2 Department of Biomedical Sciences, Institute of Tropical Medicine, Antwerp, Belgium; 3 Laboratory of Foodborne Parasitic Zoonoses, Department of Translational Physiology, Infectiology and Public Health, Faculty of Veterinary Medicine, Ghent University, Merelbeke, Belgium; 4 Department of Epidemiology and Public Health, Sciensano, Brussels, Belgium; 5 Department of Animal Health and Productions, Faculty of Agronomy and Bio-Engineering (FABI), University of Burundi, Bujumbura, Burundi

**Keywords:** *Taenia solium*, Cysticercosis, Neurocysticercosis, Burden, zDALY, Economic impact, Epilepsy, Epidemiology, Pig, Burundi

## Abstract

**Background:**

*Taenia solium* cysticercosis is a zoonotic disease that is endemic in many low- and middle-income countries where risk factors for disease transmission are present. The economic impact of cysticercosis on public health and on the pig production sector is not well known in many of those countries, including Burundi. This study aimed at estimating the burden of *T. solium* cysticercosis in Burundi including data on humans and pigs.

**Methods:**

Epidemiological and economic data were collected from literature up to July 30, 2021 and governmental and non-governmental agencies. Direct and indirect costs for neurocysticercosis (NCC)-associated epilepsy and losses due to porcine cysticercosis were estimated to assess the economic burden, while the health burden was estimated using zoonotic disability-adjusted life years (zDALYs). Different probability distributions (Uniform, Beta, Dirichlet and Gamma) were applied depending on the type of epidemiological parameter. Monte Carlo simulations and 100,000 iterations were used to calculate the 95% uncertainty interval (UI) for each parameter and perform sensitivity analyses.

**Results:**

In Burundi, 4.26 million USD (95% UI, 1,858,308–8,190,951) were estimated as economic impact due to *T. solium* cysticercosis in humans and pigs, of which 40.2% (95% UI, 10.3–75.1) of the total costs were due to NCC-associated epilepsy and 59.8% (95% UI, 24.9–89.7) of the losses due to porcine cysticercosis. The cost per NCC-associated epilepsy case was 72 USD (95% UI, 25–168), representing 30.8% of the GDP per capita in 2020. The probable incident cases and deaths for NCC-associated epilepsy were 9065 (95% UI, 2370–16,716) and 61 (95% UI, 16–114), respectively. More than 2 zDALYs (95% UI, 1.1–3.4) per thousand person-years was estimated, of which an average of 1.3 DALYs [0;0] (95% UI, 0.3–2.6) was due to NCC- associated epilepsy and 0.8 animal loss equivalents (ALEs) (95% UI, 0.3–1.5) due to porcine cysticercosis.

**Conclusions:**

This study provides evidence of a significant burden of *T. solium* cysticercosis for Burundi’s population. We urge policy makers to use these evidence-based results and put *T. solium* cysticercosis on the public health agenda of the country. This study recommends urgent action to find solutions for integrated control strategies for *T. solium* cysticercosis in Burundi.

**Supplementary Information:**

The online version contains supplementary material available at 10.1186/s12879-022-07849-7.

## Background

*Taenia solium* is a zoonotic tapeworm, highly endemic in many low- and middle-income countries in Latin America, Africa and South and South-East Asia [1–3]. The World Health Organization (WHO) in 2010 and the WHO and Food and Agriculture Organization of the United Nations (FAO) in 2014 listed *T. solium* taeniosis/cysticercosis among the most neglected tropical and food-borne diseases having significant socio-economic impacts in endemic areas [4, 5]. Many patients with neurocysticercosis (NCC) may be asymptomatic for several years before clinical signs appear [6, 7]. The main clinical manifestations due to NCC include epilepsy, severe progressive headache, hydrocephalus, dementia, and stroke, of which epilepsy is the most common neurologic disorder [6, 7]. In pigs, the parasite rarely causes clinical signs [8]. However, clinical signs such as, myositis in locomotion, chewing disorders, somnolence, loss of consciousness and seizures have been observed in some heavily infected pigs [9, 10]. The highest impact and the main economic losses due to porcine cysticercosis in the agricultural sector are due to condemnation of the pig carcass during meat inspection and the price reduction when selling infected pigs [8].

It was estimated that epilepsy affects 50 million people worldwide with more than 80% of infected people living in low-and middle-income countries [11]. Furthermore, of all epilepsy cases, 30% were estimated to be due to NCC in endemic regions [2, 3]. In Africa, the prevalence of epilepsy varies from country to country but the overall prevalence of 9.39 per 1000 was reported in sub-Saharan Africa [12, 13]. Moreover, it was estimated that more than 75% of patients with epilepsy did not have access to treatment in endemic countries [11].

Burundi is highly endemic for *T. solium* taeniosis/cysticercosis. Studies have shown the presence of the parasite in humans and pigs in three provinces of the country, Bururi, Kayanza and Ngozi [14–17]. Human cysticercosis was estimated at 4.2% by Antigen Enzyme-linked immunosorbent assay (Ag-ELISA) and 2.8% by Enzyme-linked immunoelectrotransfer blot (EITB)) in Bururi province [15]. A prevalence of 31.5% by Antibody Enzyme-linked immunosorbent assay (Ab-ELISA) and 20% by Ag-ELISA was reported in Ngozi province [16, 18], while in Kayanza province NCC-associated epilepsy was estimated at 35% by Ab-ELISA [14]. In pigs, cysticercosis was estimated at 15.5% by tongue palpation in Ngozi province [17], while a prevalence ranging from 2 to 39% by meat inspection was reported in Burundi [15].

In Burundi, pig farming plays an important role in rural communities by providing income and manure. However, poverty, lack of hygiene and inadequate knowledge of the parasite life cycle and risk behaviour may expose humans and pigs to *T. solium* cysticercosis [17]. Due to a lack of imaging tools for diagnosis (magnetic resonance imaging (MRI) and computed tomography (CT)), NCC probably remains underestimated and often undetected in health centres and hospitals in Burundi [19].

Therefore, assessing the burden of *T. solium* cysticercosis is crucial for Burundi because it allows policy makers to better understand its impact on public health and in the agricultural sector and to indicate the need for implementation of effective disease control methods. Even though data on porcine cysticercosis and NCC-associated epilepsy have been reported in Burundi [14–16, 18, 20], in-depth burden assessment of cysticercosis/NCC associated epilepsy has not yet been carried out. The present study aimed to fill this gap by quantifying the economic and health impacts of *T. solium* cysticercosis in Burundi by including both human and pig data in the burden analysis.

## Methods

### Study area and population

The study was conducted in Burundi a land-locked country in East Africa. The country is made up of 18 provinces, including 119 communes and 2910 hills.^1^ In Burundi, agriculture and livestock keeping are the pillars of the national and family economy. The agricultural sector contributes up to 95% of food supplies and 90% of export earnings. Most of the country’s population (90%) lives in rural areas [21, 22]. According to the planning of the Institut de Statistiques et d'Etudes Economiques du Burundi (ISTEEBU)/United Nations Fund for Population Activities (UNFPA)in 2013, the total population in 2020 was estimated at 11,215,758 people [23] (Table 1).

**Table 1:** Human population by age and sex categories in 2020 [23]

Age (years)	Male	Female	Total
0–4	893,872	888,554	1,782,426
5–14	1,486,390	1,514,133	3,000,523
15–44	2,459,411	2,568,012	5,027,423
45–59	470,882	481,213	952,095
60 +	218,877	234,234	453,111
All ages	5,529 432	5,686,146	11,215,578

The health system in Burundi includes 18 health provinces, 46 health districts, 955 health centres (including 555 public, 278 private and 122 denominational) and 73 hospitals (including 44 public, 20 private and 9 denominational). More than 80% of the population has access to a health structure within a radius of less than five kilometres [24]. However, the accessibility and availability of quality health care remain problematic due to the lack of qualified personnel in health facilities. In Burundi, 20,865 inhabitants share one physician (doctor), 1541 inhabitants for one nurse and 45,723 women for one midwife. In addition, half of the physicians and a third of the nurses have their activities in Bujumbura city, the country’s economic capital [24], leaving a large gap in the rural areas.

For the agricultural sector, most livestock is raised in extensive production systems [25]. The General Directorate of Livestock of the Ministry of Agriculture and Livestock coordinates livestock production and animal health services. The national veterinary laboratory in Bujumbura is the only reference veterinary laboratory that performs analyses to diagnose animal diseases. The veterinary services include a veterinary doctor in each province under the supervision of the head of the Provincial Directorate of Agriculture and Livestock and a veterinary technician at the communal and zonal level [25]. The lack of qualified personnel is observed throughout the country, which makes it difficult for farmers to access veterinary services [25]. The total number of livestock in 2017 was 1,044,649 cattle; 3,043,059 goats; 512,882 sheep; 708,867 pigs; 4,335,582 poultries and 482,260 rabbits [26]. Pig farming is currently practised in many households for manure and income, and pork consumption in urban and hill centres is high and constantly increasing. Although meat inspection is performed by veterinary inspectors in slaughterhouses, home and clandestine slaughter often occurs without veterinary inspection in Ngozi province [17].

### Cost estimation parameters for *Taenia solium* cysticercosis

To obtain data on NCC-associated epilepsy and cysticercosis in humans and pigs, previous studies carried out in Burundi for cysticercosis, taeniosis, and NCC-associated epilepsy were searched in PubMed, Web of Science and Google Scholar including both English and French languages in the search. The last online search for full titles and abstracts was on July 30th, 2021. Peer-reviewed literature available for Burundi was retrieved using the key words with Boolean operators such as “Burundi” AND (“taeniasis” OR “taeniosis” OR “tapeworm” OR “Taenia solium” OR “cysticercosis” OR “neurocysticercosis” OR “epilepsy” OR “headache” OR migraine” OR “hydrocephalus” OR “neurological disorders” OR “epilepsy/mortality” OR “cysticercosis/mortality” OR “neurocysticercosis/mortality”). The literature review was performed by the first author, with support of the last author according to Preferred Reporting Items for Systematic Reviews and Meta-Analyses (PRISMA) guidelines and followed four phases: identification, screening, eligibility and inclusion [27]. Full titles and abstracts were identified in PubMed, Web of Science and Google Scholar databases after duplicate records were removed. During the screening phase, eligibility criteria were applied and only the relevant titles and abstracts were considered. Articles with wrong country (e.g. Tanzania, Rwanda), wrong agent (e.g. *Cysticercus bovis*, *Schistosoma* spp.) and articles not directly relevant or out of the scope of the study (e.g. epilepsy and onchocerciasis in endemic areas in Africa, coenurosis in the Belgian Congo and Ruanda-Urundi) were excluded. Finally, the remaining full articles were assessed for a quantitative synthesis.

**Fig. 1:**
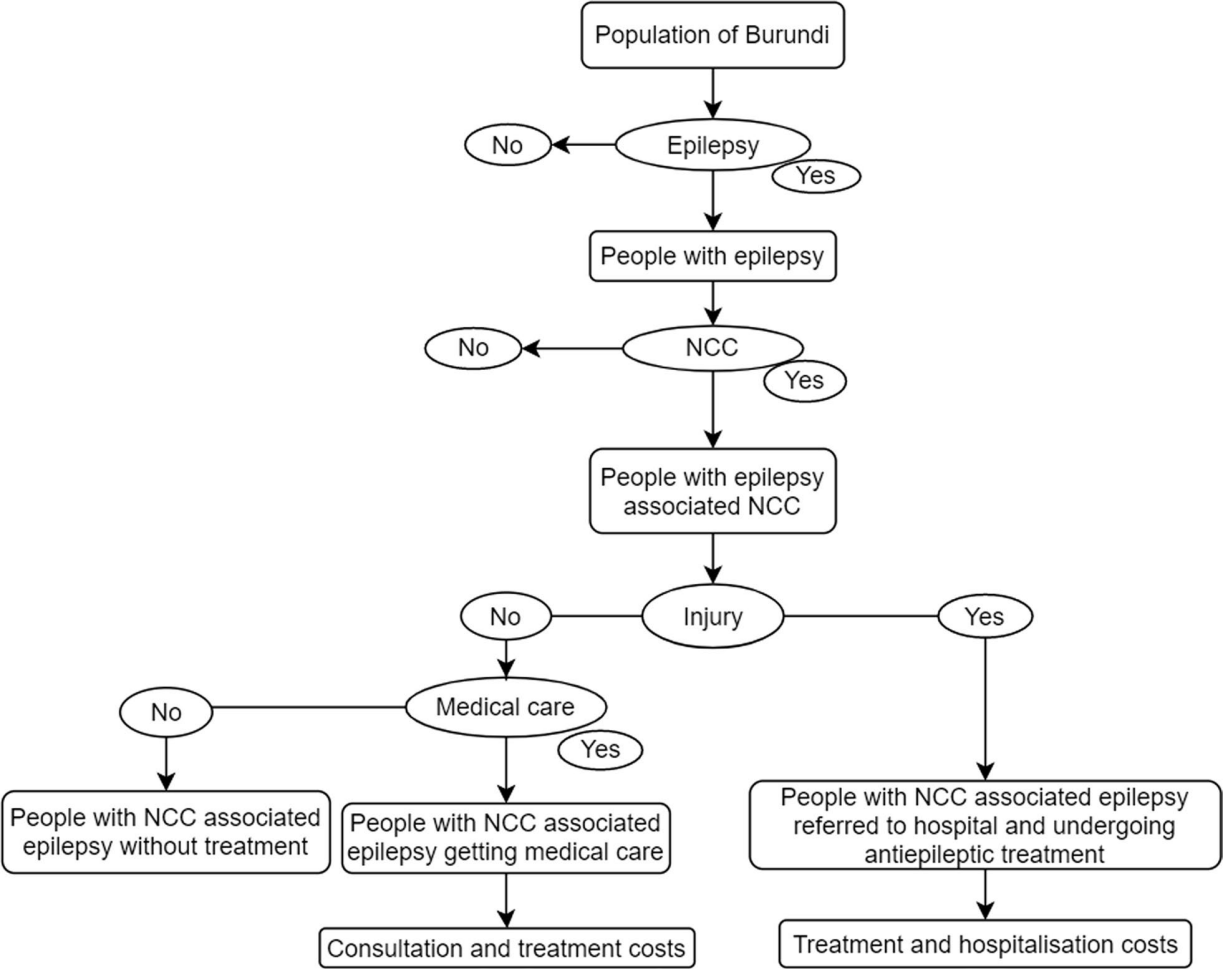
Decision tree to estimate the economic burden in Burundi

A decision tree (Fig. 1) was built to determine the proportion of the population with NCC-associated epilepsy, with or without injury and with or without treatment by the medical services [28, 29]. The patients with injury (burned by fire or injured during epileptic seizures by slipping on the floor, bicycle or car accident) were assumed to be referred to the hospital, hospitalised and got antiepileptic medication. The patients without injury were divided into: patients who sought medical care through medical consultation and patients who did not. A prevalence of 1.04% for epilepsy reported in Bururi province was used in this study [15, 30]. The prevalence of NCC-associated epilepsy in Burundi was obtained from previous studies carried out in Bururi, Kayanza and Ngozi provinces assessed using serological tests (Ag-ELISA, Ab-ELISA and EITB) [14–16, 18]. Although neuroimaging methods were not used in Burundi in these studies, serological tests were performed after looking into epilepsy history, clinical manifestations and seizure classification [15, 16]. Therefore, the probable prevalence of NCC-associated epilepsy in Burundi was considered in the economic and health burden calculations. This was based on the lower (4.9%) and upper (38.3%) values of the Ag-ELISA, as a tool for detecting active human cysticercosis [15, 18].

Epidemiological data to estimate the economic burden was collected consulting documents in governmental and non-governmental structures (Additional file 1: Table S1). The national centre in charge of epilepsy (*Centre Neuropsychiatrique de Kamenge* (CNPK)) was visited in February 2021 to collect data related to medical costs associated to epileptic patients such as, medical consultation, antiepileptic medication, and hospitalisation. In addition, the inactivity costs were quantified based on the days lost due to incapacity to work and unemployment and days spent to seek medical services. Indirect costs were estimated by applying the human capital approach based on the days of inactivity or unemployment caused by the disease. Direct costs were estimated based on the price of medical consultation, antiepileptic medication for one month and hospitalisation for one day fixed by CNPK and the Ministry of Public Health. However, consultation and treatment costs for traditional healers were not included in the assessment due to a lack of data for Burundi. We assumed that people getting medical care in Burundi received an appropriate treatment. Therefore, only the costs for people visiting official medical structures were considered in the analysis.

The losses due to porcine cysticercosis for the agricultural sector were quantified based on the porcine cysticercosis prevalence of 15.5% detected by tongue palpation [17] (Additional file 1: Table S2). Since many pig traders and butchers perform tongue palpation before buying pigs, a price reduction of up to 70–80% was applied when pigs were found infected [17]. Only a reduced selling price for pigs was considered as porcine cysticercosis has no significant effect on pig productivity [8]. In 2020, the total pig population in Burundi was estimated at 846,948 [31]. The total number of pigs sold and slaughtered in Burundi was calculated based on the FAO report in 2020 [32]. In Burundi, the pig meat production was 6000 tonnes in 2018 [32]. In addition, Levy and colleagues found that the dressed weight of an adult pig was estimated at 22.5 kg [33]. Therefore, 266,667 pigs, representing 31.5% of the total pig population were assumed as sold and slaughtered in Burundi for the year 2020.

### Health burden estimation parameters for *Taenia solium* cysticercosis

The health burden of *T. solium* in Burundi in humans and in pigs was assessed using the zoonotic disability-adjusted life years (zDALYs)) to comply with the One Health approach that involves multi-sectoral collaboration to promote community health [34, 35]. The zDALY sums the health burden in humans using DALYs, which are the sum of years of life lost due to premature mortality (YLL) and years lived with disability (YLD) to the animal loss equivalents (ALEs) for livestock [35]. The estimation of the health impact due to NCC-associated epilepsy was quantified for the year 2020. The DALY was calculated based on the probable annual number of NCC-associated epilepsy cases and the probable number of deaths from the data available from studies carried out in Burundi on human cysticercosis associated epilepsy and from available literature (Additional file 1: Table S3). Due to the non-availability of data on headache and other NCC-associated symptoms, quantification of the burden considered epilepsy only. The formulas for health burden (DALY) are reported in Eqs. 1, 2 and 3 [36, 37].

1 $${\text{DALY}} = {\text{YLL}} + {\text{YLD}}$$

2 $${\text{YLL}} = {\text{N}}*{\text{L}}$$

where ‘N’ is the number of deaths per year and ‘L’ is the standard life expectancy at the age of death in years

3 $${\text{YLD}} = {\text{I}}*{\text{DW}}*{\text{L}}$$

where ‘I’ is the number of incident cases per year, ‘DW’ is the disability weight and ‘L’ is the average duration of the disease until recovery or death.

The probable incident cases for NCC-associated epilepsy were obtained by multiplying the population of Burundi by the proportion of epilepsy and the probable proportion of NCC-associated epilepsy and then dividing by the duration of epilepsy. Stratification of disability duration according to age and sex was applied [38] (Additional file 1: Table S3). The probable annual number of deaths due to NCC-associated epilepsy were calculated by multiplying the probable incident cases for NCC-associated epilepsy by the case-fatality ratio of epilepsy (Additional file 1: Table S3). Disability weights for epilepsy used in this study were based on the findings of the Global Burden of Disease (GBD) study 2010 and 2013 [39, 40]. Life expectancy at birth from GBD 2019 was used [41].

The DALYs were calculated using the scenario of DALYs [0;0] without age weighting (K = 0) or time discounting (r = 0) and using an incidence approach [29, 42].

To estimate the health burden in the agricultural sector, a component of zDALYs, animal loss equivalents (ALEs) were estimated based on the economic losses due to porcine cysticercosis divided by the gross national per capita income (GNI) in Burundi in 2020 estimated in United States dollar (USD) [35, 43]. Data of GNI per capita were collected from the world bank figures [44]. The ALEs per thousand person-years were calculated by considering the total population of Burundi in 2020.

### Economic and health burden assessment analyses

The R software version 4.1.0 was used to perform all analyses (R Core Team, 2021). Scripts available online (https://github.com/MINANI-Salvator/Tsol-burden-Burundi) were used to estimate the health and economic impacts. Monte Carlo simulations allowed to calculate the uncertainty interval (UI) at 95% for each parameter with a mean at 50% and quantiles at 2.5% and 97.5%. Depending on the type of variable or information available for each parameter, different probability distributions were used as presented in Additional file 1: Tables S1–S4. The mean of each probability distribution was quantified based on the lowest and the highest values for a uniform distribution, two positive shape parameters α and β for a Beta distribution, a shape parameter k and a scale parameter θ for a Gamma distribution, and a vector α for a Dirichlet distribution. The number of iterations was set to 100,000 and deterministic sensitivity analyses were performed to identify the contribution of each parameter to the overall results. Sensitivity analysis functions, including standardised regression coefficients and partial correlation coefficients were set up. Then, a tornado graph that requires the ggplot2 library in R was established. Epidemiological parameters of economic and health burden were incorporated into standardised regression coefficients and partial correlation coefficients to see the level of statistical significance of the parameters, and tornado graphs were obtained indicating the parameters with the highest impacts on total DALYs and economic costs. A script for the deterministic sensitivity analysis is also available online at https://github.com/MINANI-Salvator/Tsol-burden-Burundi. In addition, a 2020 exchange rate of 1915 BIF (Burundi francs) for 1 USD (United States dollar) was used for the economic burden in humans and pigs (Additional file 1: Table S4).

## Results

### Literature retrieved in Burundi

**Fig. 2:**
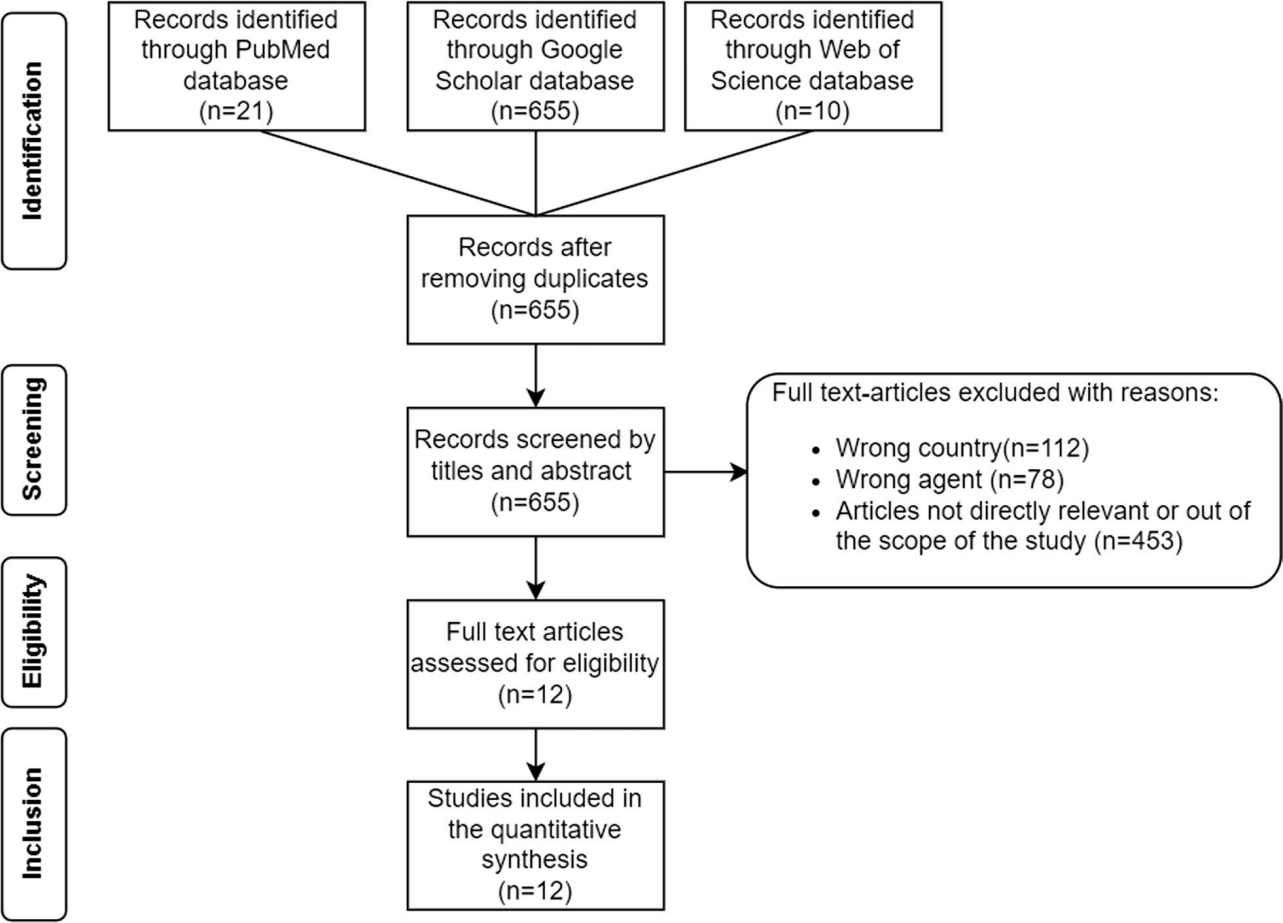
Flowchart describing the number of articles retrieved from the database

A total of 686 records were retrieved from the database (Fig. 2). After removing 31 duplicate records, 655 records were screened by titles and abstracts. Of these 643 records were excluded as not compliant with the eligibility criteria. Twelve records were retained for the quantitative synthesis. Of these, ten were articles, one was a PhD thesis and one a report. Seven articles were written in English, while three articles, the PhD thesis and the report were in French. Six articles included data on human cysticercosis and epilepsy, taeniosis and porcine cysticercosis (Table 2), while four other articles included data on epilepsy and toxocariasis [45], epilepsy associated with onchocerciasis [30], data on the economic evaluation of epilepsy in Kiremba [20], and data on knowledge, attitudes, and practices about epilepsy in rural Burundi [46]. The abstract of the report of two cases of cysticercosis in Burundi was not available online [47] and the PhD thesis described epilepsy as an unrecognised public health problem in Burundi [48].

**Table 2:** Retrieved studies in Burundi used to estimate the economic and health burden

Communes	Province	Sample size	Type of samples	Prevalence and tests	HC	T	PC	References
Buyengero and Burambi	Bururi	175	Blood	4.9% by Ag-ELISA	x			[15]
			Blood	11.7% by EITB	x			
		81	Pig carcass	2–39% by meat inspection			x	
		13,840	Stools	0–1% by microscopy		x		
Kayanza	Kayanza	84	Blood	35% by Ab-ELISA	x			[14]
		39	CSF	64% by Ab-ELISA	x			
		NA	Pig carcass	20% by meat inspection			x	
Kiremba	Ngozi	972	Blood	59.6% by Ab-ELISA	x			[16]
Kiremba	Ngozi	909	Blood	58.7% by Ab-ELISA	x			[18]
			Blood	38.3% by Ag-ELISA	x			
Kiremba	Ngozi	249	Brain	51.2% by EEG	x			[49]
			Blood	36% by Ab-ELISA	x			
Ngozi and Marangara	Ngozi	496	Pig tongue	15.5% by tongue palpation			x	[17]

*HC* human cysticercosis, *T* Taeniosis, *PC* Porcine cysticercosis, *NA* not available, *EEG* electroencephalogram

### Economic burden estimation

The economic losses due to *T. solium* cysticercosis in Burundi were estimated at 4.26 million USD (95% UI, 1,858,308–8,190,951) in 2020, of which 1.83 million USD (95% UI, 307,347–5,339,958) were associated to NCC-associated epilepsy and 2.43 million USD (95% UI, 980,778–4,575,241) to porcine cysticercosis (Table 3). Direct and indirect costs per NCC-associated epilepsy case were 72 USD (95% UI, 25–168). Direct costs (hospitalisation, medical consultation, and antiepileptic medication) related to NCC-associated epilepsy represented 33.4% (95% UI, 10.3–73.9) of the costs, while 66.6% (95% UI, 26.1–89.7) were the indirect costs (inactivity due to inability to work and unemployment). Of the total costs, 28.3% (95% UI, 3.9–63.6) were due to inactivity costs and 11.9% (95% UI, 3.5–26.4) for direct costs for human cysticercosis (40.2% (95% UI, 10.3–75.1)), respectively, while more than half (59.8% (95% UI, 24.9–89.7)) were related to porcine cysticercosis.

**Table 3:** Estimated economic costs in humans and pigs in Burundi

Type of cost	Mean of USD (95% uncertainty interval)	% of total costs (95% uncertainty interval)
*Direct costs*		
Hospital	426,702 (96,872–959,777)	10.4 (2.9–23.4)
Medical consultation	39,746 (4,650–109,676)	0.9 (0.1–2.7)
Antiepileptic medication	23,924 (6,119–46,392)	0.6 (0.2–1.2)
*Indirect costs*		
Inactivity	1,342,831 (114,076–4,605,805)	28.3 (3.9–63.6)
Pig losses	2,427,860 (980,778–4,575,241)	59.8 (24.9–89.7)
Total cost	4,261,063 (1,858,308–8,190,951)	100
Cost per NCC-associated epilepsy case	72 (25–168)	

### Health burden estimation

In Burundi, the probable annual number of incident cases and deaths due to NCC-associated epilepsy were estimated in 2020 at 9065 (95% UI, 2370–16,716) and 61 (95% UI, 16–114), respectively (Table 4). Using zDALYs in the assessment of health impacts, 2.1 zDALYs (95% UI, 1.1–3.4) per thousand person-years was estimated in Burundi, including 1.3 DALYs [0;0] (95% UI, 0.3–2.6) due to NCC- associated epilepsy and 0.8 ALEs (95% UI, 0.3–1.5) due to porcine cysticercosis. The annual number of DALYs was estimated at 14,603 (95% UI, 3715–28,624). Most DALYs (70.6% (95% UI, 62.8–76.7)) corresponded to the non-fatal burden (YLD) while the fatal burden (YLL) represented 29.4% (95% UI, 23.3–37.2) (Table 5).

**Table 4:** Estimated incident cases, incident cases without treatment and deaths due to NCC-associated epilepsy, and pigs with cysticercosis in Burundi

Estimate	Mean (95% uncertainty interval)	% of the total population (95% uncertainty interval)
Probable annual number of incident cases of NCC-associated epilepsy	9065 (2370–16,716)	0.08 (0.02–0.15)
Probable annual number of incident cases of NCC-associated epilepsy without treatment	7545 (1970–13,951)	0.07 (0.02–0.12)
Probable number of deaths due to NCC-associated epilepsy	61 (16–114)	0.0005 (0.0001–0.001)
Number of pigs with cysticercosis	131,459 (105,679–159,424)	15.5 (12.5–18.8)

**Table 5:** Estimated health burden of zoonotic disability-adjusted life years in Burundi

Estimate	Mean (95% uncertainty interval)	% Contribution to total zDALYs (95% uncertainty interval)
DALYs in humans		
YLD	10,370 (2564–21,215)	70.6 (62.8–76.7)
YLL	4233 (1097–7943)	29.4 (23.3–37.2)
Total DALYs	14,603 (3715–28,624)	100
DALY per 1000 person-years	1.3 (0.3–2.6)	58.1 (29.2–76.1)
ALEs in pigs		
ALEs	8992 (3633–16,945)	–
ALEs per 1000 person-years	0.8 (0.3–1.5)	41.9 (23.9–70.8)
zDALYs per 1000 person-years	2.1 (1.1–3.4)	100

*DALYs* disability-adjusted life years, *YLD* year lived with disability, *YLL* year of life lost, *ALEs* animal loss equivalents, *zDALYs* zoonotic disability-adjusted life years

### Sensitivity analyses

**Fig. 3:**
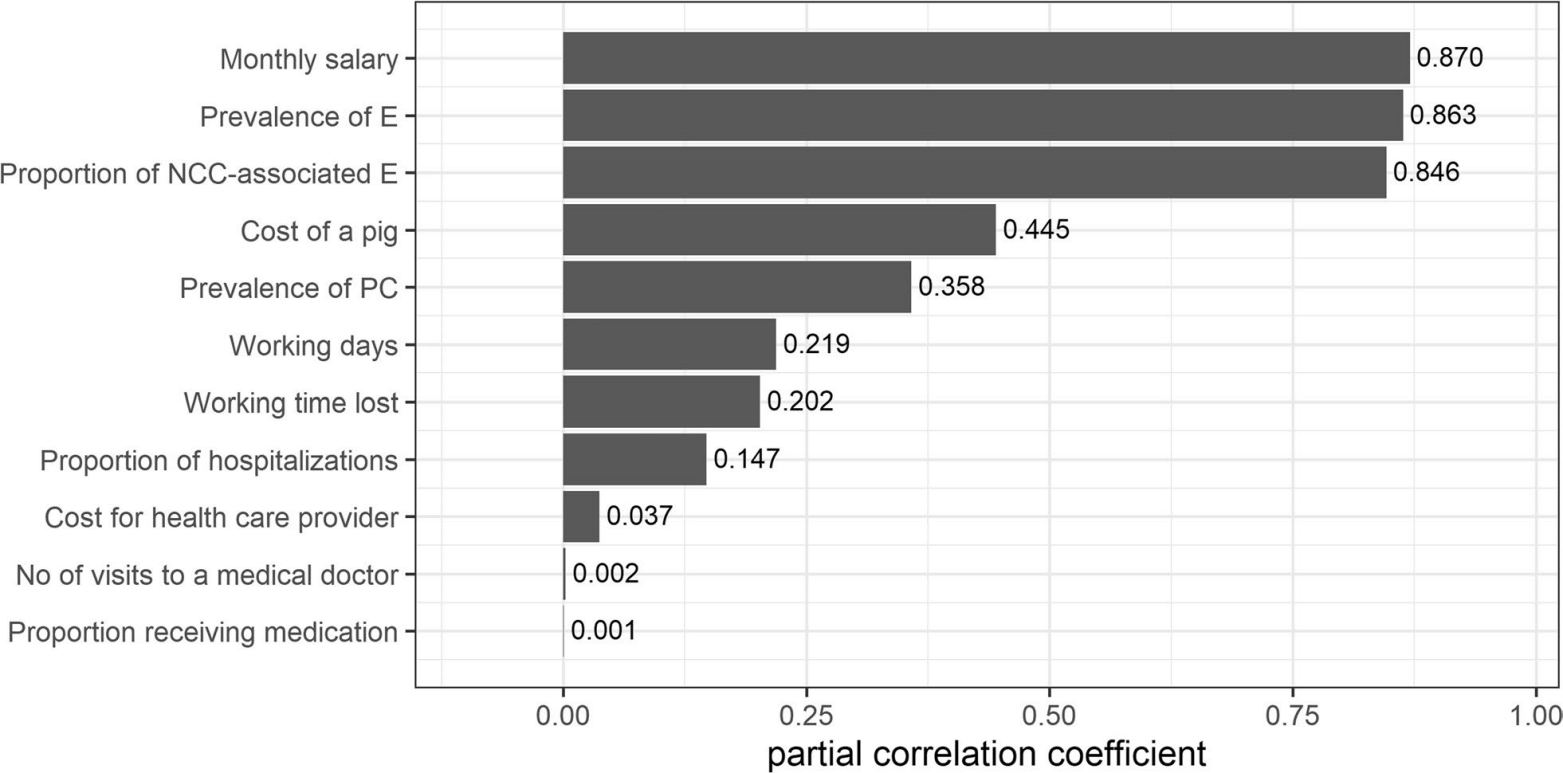
Partial correlation coefficients indicating the parameters that influence the estimated economic burden in Burundi. *E* epilepsy, *NCC* neurocysticercosis, *PC* porcine cysticercosis

**Fig. 4:**
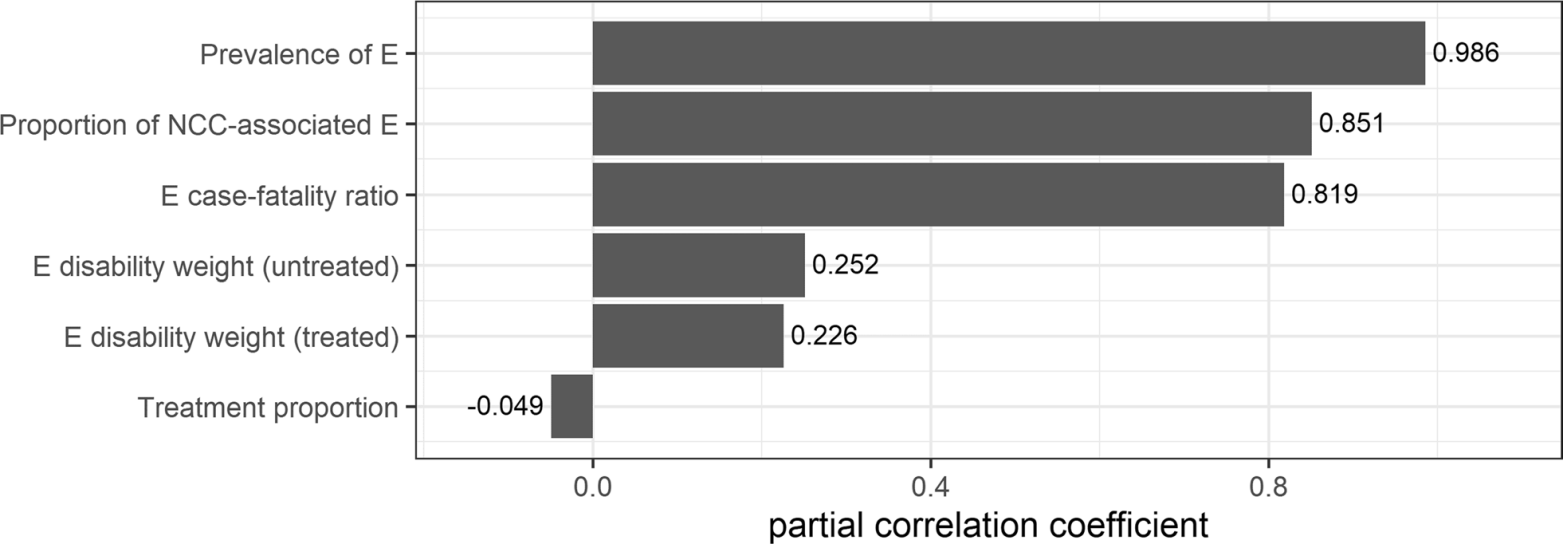
Partial correlation coefficients indicating the parameters that influence the estimated disability-adjusted life years in Burundi. *E* epilepsy, *NCC* neurocysticercosis

The partial correlation coefficients indicating the impact of different parameters on the uncertainty of the overall estimates are illustrated in the Figs. 3 and 4. Monthly salary (0.87), epilepsy prevalence (0.86), and probable proportion of NCC-associated epilepsy (0.84) were parameters contributing mostly on the economic burden (Fig. 3), while epilepsy prevalence (0.98), probable proportion of NCC-associated epilepsy (0.85) and epilepsy case-fatality ratio (0.81) contributed mostly to the health burden results (Fig. 4).

## Discussion

This study is the first in Burundi to thoroughly assess the burden of *T. solium* cysticercosis including human and pig data in the analysis. The economic losses per NCC-associated epilepsy case in this study was estimated at 72 USD per year, representing 30.8% of the gross domestic product (GDP) per capita (233.8 USD) in 2020 in Burundi [50]. This cost was lower than the average cost per NCC-associated epilepsy case reported in other studies in Africa: 106 USD estimated for the year 2012 in Tanzania [29], 738 USD in 2004 in Eastern Cape Province, South Africa (ECP) [51] and 240 USD (190 Euro) in 2008 in Cameroon [28]. In Burundi, the higher proportion of untreated cases compared to Tanzania (57.1%), ECP (29.9%) and Cameroon (22.6%), low cost of treatment, and cost of traditional healers and imaging not included in this study could be some reasons explaining the low estimated cost per NCC-associated epilepsy case. If the total costs for *T. solium* cysticercosis in humans and pigs are considered, the costs of inactivity were three times lower than those observed in Cameroon (84.9%) and one and a half times lower than in Tanzania (49.2%) [28, 29]. The large difference in the costs of inactivity was due to the lower monthly salary and the proportion of unemployment due to epilepsy in Burundi compared to Cameroon and Tanzania. This study showed that the direct costs for NCC-associated epilepsy were much lower than the indirect costs. Hospitalisation costs contributed more to the direct costs for NCC-associated epilepsy compared to medical consultation and treatment costs as many epilepsy patients stayed in the hospital for a long time with an average of 54 days [20]. In addition, 83.3% of epilepsy patients did not receive health care and antiepileptic drugs [16, 20], which is in line with the results reported by the WHO, that reported that more than 75% of epilepsy patients in low-income countries, including Burundi, and 50% in middle-income countries were not receiving medication for epilepsy [11].

When looking at the agricultural sector, the losses due to porcine cysticercosis contributed more than half of the total costs. Compared to Burundi, the pig losses contributed to the total costs for 4.7% in Cameroon, 20% in ECP and 41.4% in Tanzania [28, 29, 51]. The losses associated to porcine cysticercosis in Burundi were high because butchers or pig traders practise tongue palpation before buying pigs. In case the pigs are found to be infected with cysticerci, the selling price is reduced by up to 70–80% of the total cost [17]. In Tanzania, a 50% price reduction was used to estimate the losses for pigs while in Cameroon and ECP a 30% price reduction was taken into account [28, 29, 51]. Moreover, the prevalence of 15.5% for porcine cysticercosis in Burundi used in this study was higher compared to the prevalence of 11.7% in Tanzania and 5.6% in Cameroon, which further contributes to increased losses for the agricultural sector [17, 28, 29]. In this study, the assumption of 31.5% for pigs slaughtered was considered, nearly similar to that in Tanzania (33.3%) but much lower than those considered in Cameroon and ECP, where they assumed that all pigs raised in the country were slaughtered during the year [28, 29, 51]. If the assumption for Cameroon and ECP was applied for Burundi, the pig losses would be even more substantial, which explains the effect of the assumption used in the study. Compared to the finding of Zoli and colleagues for Burundi in 2003, an amount of 257,263 USD (218,325 Euro) was reported using the price reduction of 30% for infected pigs, which is more than nine times lower than the present finding [8]. Based on the pig population applied in this study, the number of pigs in Burundi (846,948 pigs) was nearly twice as lower than in Tanzania (1,573,080 pigs), two and a half times higher compared to in ECP (339,083 pigs) and three times higher than in Cameroon (285,606 pigs) [28, 29, 51]. Therefore, a high pig population combined with the cost of adult pig contribute more to agricultural losses due to porcine cysticercosis.

Health burden studies for NCC-associated epilepsy were carried out in many countries across the globe. The DALY metric was used in most studies to assess the health impact of *T. solium* cysticercosis. In this study, the health burden was assessed using the zDALY metric, including DALY in humans and ALEs in the agricultural sector. Our finding of 2.1 zDALYs per thousand person-years equivalent to 210 zDALYs per 100,000 person-years (130 DALYs and 80 ALEs) was much lower than that of Cameroon where 9062 zDALYs per 100,000 person-years were estimated, including 9050 DALYs [1;0.03] due to NCC-associated epilepsy and 12 ALEs due to porcine cysticercosis [35]. Compared to other foodborne parasitic diseases like cystic echinococcosis, zDALYs per 100,000 person-years estimated at 47.2 in Tunisia, 29.8 in Iran, 21.6 in Peru, 111 in Kyrgyzstan and 55 in Morocco were lower than the finding of cysticercosis in Burundi [35, 43]. In the total zDALY, ALEs due to cysticercosis contributed less than 1% in Cameroon while in Burundi they contributed 41.9% [35]. In contrast, ALEs due to cystic echinococcosis contributed more to zDALYs per 100,000 person-years in Tunisia, Iran and Morocco [35, 43].

The health burden due to NCC-associated epilepsy per thousand person-years in Burundi was higher (1.3 DALYs [0;0]) than the 0.7 DALYs [0;0] (95% UI, 0.2–1.6) found in Tanzania [29], 0.54 DALYs [0;0] (95% UI, 0.20–1.05) in Nepal [52], and 0.25 DALYs [1;0.03] (95% UI, 0.12–0.46) in Mexico [53]. The high health burden in Burundi can be due to the high prevalence of epilepsy, probable proportion of NCC-associated epilepsy and proportion of epilepsy patients without treatment. However, the DALYs per thousand person-years of this study was nearly one and a half times lower than 1.73 DALYs [0;0] (95% UI, 0.8–3.4) found in India [54], five times lower than the 6 DALYs [0;0] (95% UI, 4–8) in Mozambique [42], seven times lower than the 9 DALYs [1;0.03] (95% UI, 2.8–20.4) in Cameroon [28] and three times lower than the 3.54–3.56 DALYs [0;0.03] in Ecuador [55]. The inclusion of chronic headaches and migraine in the burden assessment could explain the high DALYs per thousand person-years in Mozambique and Ecuador, parameters not assessed in Burundi. This study showed that the probable annual number of deaths due to NCC-associated epilepsy corresponded to 0.67% of the total incident cases, which was almost similar in Mexico (0.5%) [53] but was much lower than the 6.9% in Cameroon [28] and the 1.2% in Tanzania [29]. The GBD 2016 Epilepsy Collaborators estimated 32,995 DALYs (95% UI, 18,709–53,315) for epilepsy in Burundi, almost twice the DALYs of NCC-associated epilepsy found in this study [56]. In Burundi, Mexico, and Ecuador, more than 70% of DALYs were attributed to non-fatal burden (YLD) [53, 55]. This agrees with the findings of Torgerson and colleagues, who reported that 2.8 million DALYs worldwide (95% UI, 2.14–3.61) were due to human cysticercosis, with the main proportion attributed to YLD [57]. In contrast, more than 80% of DALYs were attributed to YLL in both Cameroon and India [28, 54]. The high number of YLD could be explained by the fact that many epilepsy patients did not consult official medical services, which increased the DALY estimate [16]. Epilepsy patients in Burundi believed that epilepsy was contagious, incurable and caused by evil spirits, hence the lack of interest in seeking treatment in medical facilities [13, 46]. In addition, wrong beliefs of some people about epilepsy transmission through saliva and urine emitted by patients during seizure increase the stigma and discrimination of these patients in the communities [46].

The results of the sensitivity analysis are in line with the findings in Tanzania, where epilepsy prevalence, proportion of NCC-associated epilepsy, together with epilepsy case-fatality ratio and monthly salary appear to be the most significant parameters [29].

Our study has some limitations. The prevalence of epilepsy used in the study was reported in 1997, which might not correspond to the current situation in Burundi. However, a prevalence of epilepsy of 9.39 per 1000 was reported in Africa, which is almost similar to that used in our estimate for Burundi [13]. In Burundi, cases of human cysticercosis have been diagnosed using serological tests only (EITB, Ab-ELISA, Ag-ELISA) [14–16, 18] because neuroimaging tools such as, MRI and CT scans were not available to confirm serological test results [18, 58, 59]. Although severe progressive headache is a clinical manifestation of NCC [6, 60], it was not included in this study because no studies looking into headache were performed in Burundi. Furthermore, there are other clinical signs such as hydrocephalus, stroke and dementia that were not included in the DALYs estimates [6, 7]. Moreover, the lack of data on traditional healers in Burundi from the available literature was also a challenge for estimating direct costs due to NCC-associated epilepsy. Traditional healers exist in Burundi and sell herbal products in the market or at home [61]. Nevertheless, the costs for an epilepsy consultation and medication for traditional healers are missing as many traditional healers work clandestinely and consultation at home is not regulated by the law although it is practised routinely.

For the agricultural sector, the lack of epidemiological studies on porcine cysticercosis in different areas of the country was a challenge, whereby only the prevalence estimated in one province could be used for this study [17]. Therefore, the estimated prevalence in pigs appeared to be higher than in other countries where the studies on the burden of *T. solium* cysticercosis were performed. To get the number of pigs slaughtered in Burundi, an assumption was made based on pork production. It would be useful for the livestock department to report slaughtered pigs annually, as meat inspection seems to be practised for all livestock species that are slaughtered in official abattoirs. However, this would not take into account backyard slaughtering of pigs. The losses due to the condemnation of infected pigs were not considered in this study as no report was available. Including the value of condemned pigs is likely to significantly increase the financial loss caused by porcine cysticercosis.

## Conclusions

This study is the first to assess the health and economic impacts due to *T. solium* cysticercosis in Burundi. The study showed that *T. solium* cysticercosis is a serious threat for the public health and agricultural sectors in Burundi causing 2.1 zDALYs per thousand person-years, a loss of 1.83 million USD per year as a result of NCC-associated epilepsy, and a loss of 2.43 million USD due to porcine cysticercosis. To reduce the burden due to *T. solium* cysticercosis, urgent action following a One Health approach should be implemented to find solutions for integrated control strategies for *T. solium* cysticercosis in Burundi. Further studies to update information on epilepsy and cysticercosis are needed. Furthermore, data on other symptoms/costs that contribute to the burden such as severe progressive headache, hydrocephalus, vision loss and costs for traditional healers should be collected, as current estimates are likely to be an underestimation. We urge policy makers to use these evidence-based results and put *T. solium* cysticercosis on the public health agenda of the country.

## Supplementary Information

**Additional file 1:  Table S1.** Epidemiological parameters used to estimate the economic burden in humans. **Table S2.** Epidemiological parameters used to estimate the economic losses in pigs. **Table S3.** Parameters used to estimate the health burden. **Table S4.** Economic parameters used to estimate the economic burden.

## Data Availability

The datasets used and/or analysed during the current study are available from the corresponding author on reasonable request.

## Abbreviations

Ab-ELISA: Antibody enzyme-linked immunosorbent assay
Ag-ELISA: Antigen enzyme-linked immunosorbent assay
ALE: Animal loss equivalent
BIF: Burundi francs
CNPK: Centre Neuropsychiatrique de Kamenge
CT: Computed tomography
DALY: Disability-adjusted life year
ECP: Eastern Cape Province, South Africa
EITB: Enzyme-linked immunoelectrotransfer blot
FAO: Food and Agriculture Organization of the United Nations
GBD: Global burden of disease
GDP: Gross domestic product
GNI: Gross national per capita income
ISTEEBU: Institut de Statistiques et d'Etudes Economiques du Burundi
MRI: Magnetic resonance imaging
NCC: Neurocysticercosis
PRISMA: Preferred Reporting Items for Systematic Reviews and Meta-Analyses
UI: Uncertainty interval
USD: United States Dollar
WHO: World Health Organization
YLD: Years lived with disability
YLL: Years of life lost
zDALY: Zoonotic disability-adjusted life year

## Footnotes

1 Hill is the third level of administrative division in Burundi after the provinces and communes. Each commune contains a large number of hills. Hill is comparable to villages in municipalities.
