# Supplementary material for: Assessing the burden of Taenia solium cysticercosis in Burundi, 2020
Source: BMC Infect Dis. 2022 Nov 14;22:851. doi: 10.1186/s12879-022-07849-7 (PMC9661784; doi:10.1186/s12879-022-07849-7)
Supplement: Supplementary file 1 — Additional file 1: Table S1. Epidemiological parameters used to estimate the economic burden in humans. Table S2. Epidemiological parameters used to estimate the economic losses in pigs. Table S3. Parameters used to estimate the health burden. Table S4. Economic parameters used to estimate the economic burden. [file 12879_2022_7849_MOESM1_ESM.docx]

**Additional file 1**

**Table S1: Epidemiological parameters used to estimate the economic burden in humans**

| **Parameter** | **Value (95% uncertainty interval)** | **Distribution** | **Reference** |
| --- | --- | --- | --- |
| Population of the study zone (2020) | 11,215,578 | Fixed | [23] |
| Epilepsy prevalence | 0.010 (0.008-0.013) | Beta (85,8046) | [15,30] |
| Probable proportion of epilepsy associated with neurocysticercosis | 0.216 (0.057-0.375) | Uniform (0.049,0.383) | [15,18] |
| People with epilepsy and with injury referred to the hospital | 0.080 (0.054-0.110) | Beta (28,324) | [20] |
| People with epilepsy getting medical care | 0.168 (0.130-0.209) | Beta (59,293) | [34] |
| People with epilepsy without treatment | 0.833 (0.791-0.872) | Beta (270,54) | [16] |
| Number of visits to a doctor in case of epilepsy (per year) | 6.5 (1.3-11.73) | Uniform (1,12) | CNPK, Ntunzwenimana V., personal communication |
| Length of stay in a hospital | 54 (3.6-104.3) | Uniform (1,107) | [20] |
| People with epilepsy prescribed phenobarbital | 0.133 (0.038-0.274) | Dirichlet (4) | CNPK, unpublished data |
| People with epilepsy prescribed carbamazepine | 0.367 (0.207-0.543) | Dirichlet (11) | CNPK, unpublished data |
| People with epilepsy prescribed phenytoin | 0.067 (0.008-0.177) | Dirichlet (2) | CNPK, unpublished data |
| People with epilepsy prescribed valproate | 0.433 (0.264-0.612) | Dirichlet (13) | CNPK, unpublished data |
| Loss of working time due to epilepsy (days per year) | 12 (3.8-24.9) | Gamma (4.8, 0.4) | CNPK, Ntunzwenimana V., personal communication |
| Unemployed due to epilepsy | 0.162 (0.125-0.202) | Beta (57,295) | [34] |
| % of the population: |  |  |  |
| Economically active | 0.5506 | Fixed | [23] |
| Not economically active | 0.4264 | Fixed | [23] |
| Unemployed | 0.0228 | Fixed | [23] |
| Working days per year | 266 (222.3-309.7) | Uniform (220,312) | [29] |

The column of 95% uncertainty interval represents the mean of the parameter or the distribution at 50% (0.5) with the values in parentheses representing the quantiles at 2.5% (0.025) and 97.5% (0.975). The values in parentheses in the distribution column represent the lowest and the highest values for a uniform distribution (proportion of epilepsy associated with NCC: min: 4.9% (0.049), max: 38.3% (0.383)); two positive shape parameters α and β for a Beta distribution (epilepsy prevalence: α=85, β=8046); a shape parameter k and a scale parameter θ for a Gamma distribution (loss of working time: k=4.8, θ=0.4); and a vector α for a Dirichlet distribution (antiepileptic drugs: α=(4,11,2,13)). To see all the calculations made, please click on the link (<https://github.com/MINANI-Salvator/Tsol-burden-Burundi>).

**Table S2: Epidemiological parameters used to estimate the economic losses in pigs**

| **Parameter** | **Value (95% uncertainty interval)** | **Distribution** | **Reference** |
| --- | --- | --- | --- |
| Pig population in the study area | 846,948 | Fixed | [31] |
| Prevalence of porcine cysticercosis | 0.155 (0.125-0.188) | Beta (77,419) | [17] |
| Value reduction of infected pork | 0.75 (0.702-0.798) | Uniform (0.7,0.8) | [17] |
| Proportion of pigs sold per year | 0.315 | Fixed | Assumption based on pork production [32] |

The column of 95% uncertainty interval represents the mean of the parameter or the distribution at 50% (0.5) with the values in parentheses representing the quantiles at 2.5% (0.025) and 97.5% (0.975). The values in parentheses in the distribution column represent the lowest and the highest values for a uniform distribution (proportion of value reduction of infected pork: min: 70% (0.7), max: 80% (0.8)) and two positive shape parameters α and β for a Beta distribution (prevalence of porcine cysticercosis: α=77, β=419 ). To see all the calculations made, please click on the link (<https://github.com/MINANI-Salvator/Tsol-burden-Burundi>).

**Table S3: Parameters used to estimate the health burden**

| **Parameter** | **Value (95% uncertainty interval)** | **Distribution** | **Reference** |
| --- | --- | --- | --- |
| Epilepsy prevalence | 0.010 (0.008-0.013) | Beta (85,8046) | [15,30] |
| Probable proportion of epilepsy associated with neurocysticercosis | 0.216 (0.057-0.375) | Uniform (0.049,0.383) | [15,18] |
| Proportion of people with epilepsy receiving proper treatment | 0.168 (0.130-0.209) | Beta (59,293) | [34] |
|  |  |  |  |
| Epilepsy case-fatality ratio | 0.007 (0.006-0.008) | Beta (279,41296) | [45] |
| Average duration of disability (years) in males: |  |  |  |
| 0 and 4 years | 1.4 | Fixed | [46] |
| 5 and 14 years | 2.0 | Fixed | [46] |
| 15 and 44 years | 3.6 | Fixed | [46] |
| 45 and 59 years | 2.8 | Fixed | [46] |
| > 60 years | 1.6 | Fixed | [46] |
| Average duration of disability (years) in females: |  |  |  |
| 0 and 4 years | 1.6 | Fixed | [46] |
| 5 and 14 years | 3.1 | Fixed | [46] |
| 15 and 44 years | 5.9 | Fixed | [46] |
| 45 and 59 years | 6.0 | Fixed | [46] |
| > 60 years | 2.8 | Fixed | [46] |
| Disability weight for people with: |  |  |  |
| Epilepsy (treated) | 0.328 (0.217-0.439) | Uniform (0.211,0.445) | [39] |
| Epilepsy (untreated) | 0.426 (0.286-0.565) | Uniform (0.279,0.572) | [39] |

The column of 95% uncertainty interval represents the mean of the parameter or the distribution at 50% (0.5) with the values in parentheses representing the quantiles at 2.5% (0.025) and 97.5% (0.975). The values in parentheses in the distribution column represent the lowest and the highest values for a uniform distribution (proportion of epilepsy associated with NCC: min: 4.9% (0.049), max: 38.3% (0.383); epilepsy treated (disability weights between 0 to 1): min: 0.211, max: 0.445) and two positive shape parameters α and β for a Beta distribution (epilepsy prevalence: α=85, β=8046; epilepsy case fatality ratio: α=279, β=41296). To see all the calculations made, please click on the link (<https://github.com/MINANI-Salvator/Tsol-burden-Burundi>).

**Table S4: Economic parameters used to estimate the economic burden**

| **Parameter** | **Value or range of values (BIF*)** | **Distribution** | **Reference** |
| --- | --- | --- | --- |
| Monthly salary (BIF) | 90,000 (12,342- 243,241) | Gamma (2.16, 2.4e-05) | Assumption based on data from the Ministry of Employment |
| Cost of a visit to a physician (hospital) (BIF) | 3,000 | Fixed | CNPK, unpublished data |
| Cost of one day at the hospital (BIF) | 7,500 (5,127-9,874) | Uniform (5000,10000) | CNPK, unpublished data |
| Cost of antiepileptic drugs: |  |  |  |
| Carbamazepine (1 month of treatment) (BIF) | 7,200 | Fixed | CNPK, unpublished data |
| Phenobarbital (1 month of treatment) (BIF) | 4,650 | Fixed | CNPK, unpublished data |
| Phenytoin (1 month of treatment) (BIF) | 1,120 | Fixed | CNPK, unpublished data |
| Valproate (1 month of treatment) (BIF) | 10,150 | Fixed | CNPK, unpublished data |
| Average value of an adult pig (BIF) | 150,000 (62,442- 274,364) | Gamma (7.5, 5e-05) | Veterinary services (2020), unpublished data |
| Gross national per capita income (GNI) in BIF | 517,050 | Fixed | [44] |

*Based on 2020 exchange rate 1915BIF=1USD

The column of 95% uncertainty interval represents the mean of the parameter or the distribution at 50% (0.5) with the values in parentheses representing the quantiles at 2.5% (0.025) and 97.5% (0.975). The values in parentheses in the distribution column represent the lowest and the highest values for a uniform distribution (cost of one day at the hospital: min: 5000, max: 10000) and a shape parameter k and a scale parameter θ for a Gamma distribution (monthly salary: k=2.16, θ=2.4e-05; average value of an adult pig: k=7.5, θ=5e-05). To see all the calculations made, please click on the link (<https://github.com/MINANI-Salvator/Tsol-burden-Burundi>).
